# Supplementary material for: Natural Killer Cell Activation Receptor NKp30 Oligomerization Depends on Its N-Glycosylation
Source: Cancers (Basel). 2020 Jul 21;12(7):1998. doi: 10.3390/cancers12071998 (PMC7409301; doi:10.3390/cancers12071998)
Supplement: Supplementary file 1 [file cancers-12-01998-s001.pdf]

cancers

MDPI

Article

Natural Killer Cell Activation Receptor NKp30 Oligomerization Depends on its *N*-Glycosylation

Ondřej Skořepa, Samuel Pazicky, Barbora Kalousková, Jan Bláha, Celeste Abreu, Tomáš Ječmen, Michal Rosůlek, Alexander Fish, Arthur Sedivy, Karl Harlos, Jan Dohnálek, Tereza Skálová, Ondřej Vaněk

Supplementary Materials

Supplementary Methods

1. Mass spectrometry

Disulfide bonds in produced proteins were determined according to the previously published protocol [43]. Briefly, proteins were separated by SDS-PAGE, deglycosylated by ENDO Hf (New England Biolabs, Ipswich, MA, USA), and digested by trypsin (Sigma, St. Louis, MT, USA) under non-reducing conditions in the presence of 200 μM cystamine. The peptide mixtures were desalted on reversed phase trap column (Acclaim PepMap 100, C18, 0.1 × 20 mm, 5 μm) and separated on reversed phase analytical column (Acclaim PepMap 100, C18, 0.075 × 150 mm, 3 μm; both columns Thermo Scientific, Waltham, MA, USA) connected directly to solariX XR 12T FT-ICR mass spectrometer (Bruker Daltonics, Billerica, MA, USA) using an electrospray ion source. Data were acquired using SolarixControl 3.0.0 and processed with DataAnalysis 4.2. The disulfide bonds and saccharide moieties were identified using the Links software [44].

Glycosylation on the NKp30_Stalk protein expressed in HEK293S GnTI$^-$ cell line was characterized using an ESI FT-ICR MS according to previously published protocol [45]. Briefly, 10 μg of the protein was desalted on protein microtrap column (C4 phase, Michrom Bioresources, Auburn, CA, USA) according to the manufacturer instructions, and eluted in 50 μL of 80% acetonitrile/1% acetic acid directly to solariX XR 12T FT-ICR mass spectrometer (Bruker Daltonics, Billerica, MA, USA) using an electrospray ion source. Acquisition of mass spectra was done in the positive mode with the following parameters: the *m/z* range 250–3000, 1 M data points transient, 0.2 s ion accumulation, 4 scans accumulation. Acquired data were interpreted by DataAnalysis 4.2. A glycosylation pattern of NKp30_Stalk protein was verified by qCID MSMS analysis of isolated most abundant two times glycosylated form at *m/z* 1105.43, z = 16 (Figure S2). Acquisition of MS-MS spectra was done with 0.8 s ion accumulation and eight scans accumulation at the same *m/z* range and data points transient as MS spectra.

For glycopeptide analysis of B7-H6 expressed in HEK293T cell line the protein sample was diluted by 50 mM ammonium bicarbonate to 2 μg/μl, the disulfide bonds were reduced 60 min at 60 °C by 5 mM dithiothreitol and alkylated 60 min at room temperature in the dark by 15 mM iodoacetamide. The protein was digested overnight at 37 °C by trypsin at enzyme-to-protein ratio of 1:20 (*w*/*w*). Peptides were desalted on a peptide microtrap column (Michrom Bioresources, Auburn, CA, USA), lyophilized, reconstituted in aqueous 0.1% formic acid, and loaded onto reversed phase column (ZORBAX Eclipse Plus C18, 2.1 × 100 mm, 1.8 μm; Agilent Technologies, Santa Clara, CA, USA). The mobile phase consisted of 0.1% formic acid in water (solvent A) and 0.1% formic acid in acetonitrile (solvent B). The separation was carried out at 0.25 mL/min flow rate, the column temperature was 40 °C, and the following gradient was used (min/% B): 0/3, 1/6, 21/30, 26/80, 28/80, 29/3, 30/3. The measurement was performed on qTOF maXis mass spectrometer (Bruker Daltonics, Billerica, MA, USA) using an electrospray ion source. Acquisition of mass spectra was done in positive mode with following parameters: *m/z* range 150–2200, with CID fragmentation (precursor *m/z* range 600–2200, charge range 2–5, collision energy 70 eV). DataAnalysis 4.4 and ProteinScape 4

were used for data evaluation and assigned tandem mass spectra of glycopeptides were verified manually.

2. SEC-SAXS Measurements

Size exclusion chromatography–small-angle X-ray scattering (SEC-SAXS) data for NKp30_LBD, NKp30_Stalk, B7-H6 and its complex with NKp30_Stalk were collected at the Diamond Light Source (Didcot, Oxfordshire, UK) at beamline 21 using an Agilent 1200 HPLC system with a 2.4 mL Superdex 200 PC 3.2/30 column (GE Healthcare, Chicago, IL, USA) and a Pilatus P3-2M detector at 12.4 keV of radiation and at 4.014 m of sample-to-detector distance. NKp30_LBD and NKp30_Stalk were expressed in HEK293T cell line with wild-type human N-glycosylation, whereas the B7-H6 ectodomain was expressed in HEK293S GnTI$^-$ cell line with uniform Asn-GlcNAc$_2$Man$_5$ glycans. Proteins were diluted in 10 mM HEPES pH 7.5, 150 mM NaCl, 10 mM NaN_3. Complex was mixed at a 1:1 molar ratio. The data were collected at 293 K for buffer and protein samples at a loading concentration of 5 mg/mL for NKp30_LBD and B7-H6, and at 10 mg/mL for NKp30_Stalk and for its equimolar mixture with B7-H6. The data in selected intervals (monomeric and oligomeric fractions of NKp30_Stalk, as well as of its mixture with B7-H6, were analyzed separately) were solvent-subtracted in SCÅTTER (developed by Robert Rambo at the Diamond Light Source, http://www.bioisis.net/tutorial/9), and merged and characterized using the ATSAS64 package [37]. As a proof of the data quality, scattering and Guinier plots, for all data ranges, are shown in Figure S5. Using the pair distance distribution functions P(r), twenty ab initio structure models were calculated with DAMMIF 2.7.265 for each of the data intervals. The models were compared using the DAMSEL command and averaged using DAMAVER 5.0 (r10553). The final models were visualized in UCSF Chimera [38] and are shown in Figure S6

Table S1. Mass spectrometry analysis of disulfide bonds in the prepared recombinant proteins.

Exp. Mass	Thr. Mass	Error	Peptide	Chain	Modification	Disulfide Bond
				B7-H6 wild-type (deglycosylated)		
2633.251	2633.251	0.21	B7-H6_WT (122-130) [A]-B7-H6_WT (202-214) [B]	CEVVVTPLK-NMDGTFNVTSCLK	GlcNAc (208)	(122-212)
2879.427	2879.428	0.14	B7-H6 _WT (122-130) [A]-B7-H6_WT (215-231) [B]	CEVVVTPLK-LNSSQEDPGTVYQCVVR		(122-228)
3743.666	3743.672	0.70	B7-H6_WT (202-214) [A]-B7-H6_WT (215-231) [B]	NMDGTFNVTSCLK-LNSSQEDPGTVYQCVVR	GlcNAc (208); GlcNAc (216)	(212-228)
4086.855	4086.855	0.09	B7-H6 _WT (122-130) [A]-B7-H6_WT (156-179) [B]	CEVVVTPLK-ENEDKYMCESSGFYPEAINITWEK	GlcNAc (174)	(122-163)
4748.019	4748.019	0.06	B7-H6 _WT (156-179) [A]-B7-H6_WT (202-214) [B]	ENEDKYMCESSGFYPEAINITWEK-NMDGTFNVTSCLK	GlcNAc (174); GlcNAc (208)	(163-212)
5197.274	5197.276	0.33	B7-H6_WT (156-179) [A]-B7-H6_WT (215-231) [B]	ENEDKYMCESSGFYPEAINITWEK-LNSSQEDPGTVYQCVVR	GlcNAc (174); GlcNAc (216)	(163-228)
				B7-H6 C212S mutant (HEK293S GnTI-)		
5181.28	5181.28	−0.9	B7-H6_C212S (156, 179) [A]-B7-H6_C212S (215, 231) [B]	ENEDKYMCESSGFYPEAINITWEK-LNSSQEDPGTVYQCVVR	GlcNAc (A.174); GlcNAc (B.216)	(163-228)
4978.20	4978.20	−0.4	B7-H6_C212S (156, 179) [A]-B7-H6_C212S (215, 231) [B]	ENEDKYMCESSGFYPEAINITWEK-LNSSQEDPGTVYQCVVR	GlcNAc (A.174) ǀ GlcNAc (B.216)	(163-228)
6178.85	6178.84	1.03	B7-H6_C212S (147, 179) [A]-B7-H6_C212S (215, 231) [B]	LLLDQVGMKENEDKYMCESSGFYPEAINITWEK-LNSSQEDPGTVYQCVVR	GlcNAc (A.174); GlcNAc (B.216)	(163-228)
6071.96	6071.95	1.65	B7-H6_C212S (28, 68) [A]-B7-H6_C212S (122, 130) [B]	VEMMAGGTQITPLNDNVTIFCNIFYSQPLNITSMGITWFWK-CEVVVTPLK	GlcNAc (A.43), GlcNAc (A.57)	(48-122)
4566.03	4566.03	0.03	B7-H6_C212S (161, 179) [A]-B7-H6_C212S (215, 231) [B]	YMCESSGFYPEAINITWEK-LNSSQEDPGTVYQCVVR	GlcNAc (A.174); GlcNAc (B.216)	(163-228)
5975.77	5975.76	1.15	B7-H6_C212S (147, 179) [A]-B7-H6_C212S (215, 231) [B]	LLLDQVGMKENEDKYMCESSGFYPEAINITWEK-LNSSQEDPGTVYQCVVR	GlcNAc (A.174) ǀ GlcNAc (B.216)	(163-228)
4362.95	4362.95	0.04	B7-H6_C212S (161, 179) [A]-B7-H6_C212S (215, 231) [B]	YMCESSGFYPEAINITWEK-LNSSQEDPGTVYQCVVR	GlcNAc (A.174) ǀ GlcNAc (B.216)	(163-228)
9554.39	9554.38	1.03	B7-H6_C212S (28, 68) [A]-B7-H6_C212S (109, 130) [B]	VEMMAGGTQITPLNDNVTIFCNIFYSQPLNITSMGITWFWK-LPGIQLEEAGEYRCEVVVTPLK	$GlcNAc_2Man_5$ (A.43), $GlcNAc_2Man_5$ (A.57)	(48-122)
4978.20	4978.20	0.58	B7-H6_C212S (156, 179) [A]-B7-H6_C212S (215, 231) [B]	ENEDKYMCESSGFYPEAINITWEK-LNSSQEDPGTVYQCVVR	GlcNAc (A.174) ǀ GlcNAc (B.216)	(163-228)
4775.12	4775.12	0.1	B7-H6_C212S (156, 179) [A]-B7-H6_C212S (215, 231) [B]	ENEDKYMCESSGFYPEAINITWEK-LNSSQEDPGTVYQCVVR		(163-228)

7207.98	7207.97	1.58	B7-H6_C212S (156, 179) [A]-B7-H6_C212S (215, 231) [B]	ENEDKYMCESSGFYPEAINITWEK-LNSSQEDPGTVYQCVVR	$GlcNAc_2Man_5$ (A.174); $GlcNAc_2Man_5$ (B.216)	(163-228)
7527.69	7527.69	0.61	B7-H6_C212S (28, 68) [A]-B7-H6_C212S (109, 130) [B]	VEMMAGGTQITPLNDNVTIFCNIFYSQPLNITSMGITWFWK-LPGIQLEEAGEYRCEVVVTPLK	GlcNAc (A.43), GlcNAc (A.57)	(48-122)
9121.55	9121.54	1.15	B7-H6_C212S (28, 68) [A]-B7-H6_C212S (109, 146) [B]	VEMMAGGTQITPLNDNVTIFCNIFYSQPLNITSMGITWFWK-LPGIQLEEAGEYRCEVVVTPLKAQGTVQLEVVASPASR	GlcNAc (A.43), GlcNAc (A.57)	(48-122)
				NKp30_Stalk (deglycosylated)		
3292.49	3292.50	−1.7	NKp30_Stalk (29, 47) [A]-NKp30_Stalk (100, 94) [B]	TLEGSSAFLPCSFNASQGR-GHDASIYVCR	GlcNAc (A.42)	(39-108)
3292.50	3292.50	0.79	NKp30_Stalk (29, 47) [A]-NKp30_Stalk (100, 109) [B]	TLEGSSAFLPCSFNASQGR-GHDASIYVCR	GlcNAc (A.42)	(39-108)
3292.50	3292.50	0.78	NKp30_Stalk (29, 47) [A]-NKp30_Stalk (100, 109) [B]	TLEGSSAFLPCSFNASQGR-GHDASIYVCR	GlcNAc (A.42)	(39-108)
3089.42	3089.42	−0.3	NKp30_Stalk (29, 47) [A]-NKp30_Stalk (100, 109) [B]	TLEGSSAFLPCSFNASQGR-GHDASIYVCR		(39-108)
3662.69	3662.70	−0.8	NKp30_Stalk (29, 47) [A]-NKp30_Stalk (97, 109) [B]	TLEGSSAFLPCSFNASQGR-DVRGHDASIYVCR	GlcNAc (A.42)	(39-108)

The measured and predicted masses of the identified cystic dipeptides are given below for B7-H6_WT, B7-H6_C212S, and NKp30_Stalk expression constructs. For the wild-type B7-H6 construct we confirmed mismatch disulfide pairing with an odd cysteine 212. This mismatch was completely solved by site-directed mutagenesis (C212S) that resulted in correct disulfide pairing, i.e., only the expected disulfides between cysteines 48–122 and 163–228 were identified for the B7-H6_C212S mutant protein. For NKp30 the expected disulfide pairing between cysteines 39–108 was confirmed as well. Glycosylation of NKp30_Stalk construct produced in HEK293S GnTI$^-$ cells was analyzed by mass spectrometry. Three *N*-glycosylation sites are predicted from its amino acid sequence. By direct protein mass spectrometry, measured mass of the intact protein was majorly 17671.87 Da which suggested that the protein has two occupied *N*-glycosylation sites (Figure S1). Minor form with higher mass corresponding to three occupied *N*-glycosylation sites was also detected, albeit at much lower intensities in the mass spectrum, therefore it should be significantly less abundant in the sample. By LC MS analysis of peptide fragments, Asn42 and Asn121 were detected as fully occupied, while Asn68 was just partially glycosylated. Fragmentation spectra further confirmed the expected Asn-GlcNAc$_2$Man$_5$ composition of *N*-glycan chains produced by the HEK293S GnTI$^-$ cell line (Figure S2).

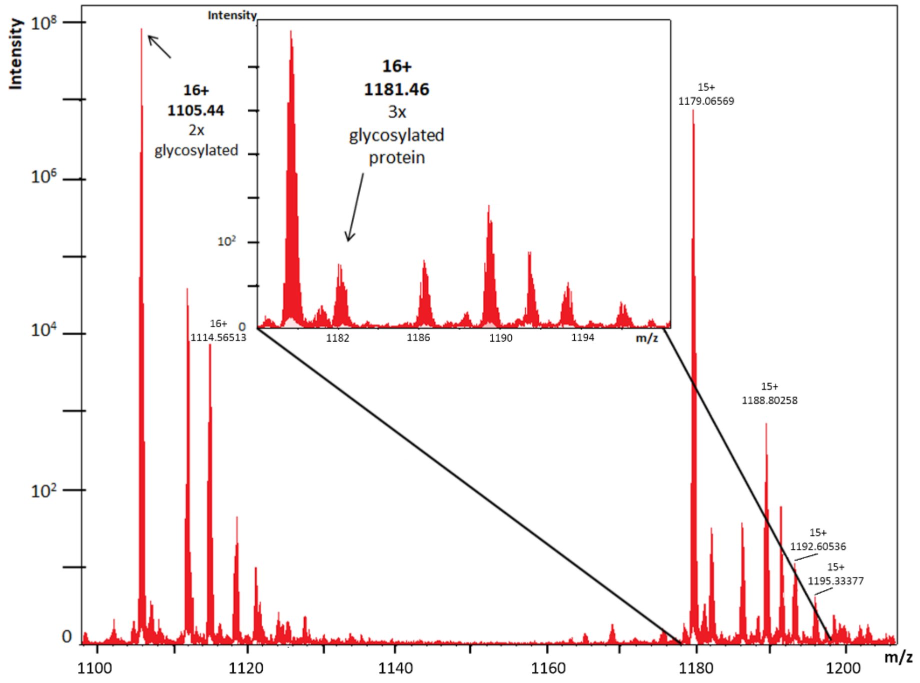

Figure S1. Direct mass spectrometry of NKp30_Stalk with uniform glycans produced by the HEK293S GnTI$^-$ cell line. Peak with the observed *m*/*z* value of 1105.44 corresponds to the 16× charged protein with two occupied *N*-glycosylation sites. Intensity of this peak is at 10^8 order of magnitude. Peak with the observed *m*/*z* value of 1181.46 and intensity at 10^2 corresponds to the 16× charged protein with three occupied *N*-glycosylation sites which is thus considerably less abundant in the sample.

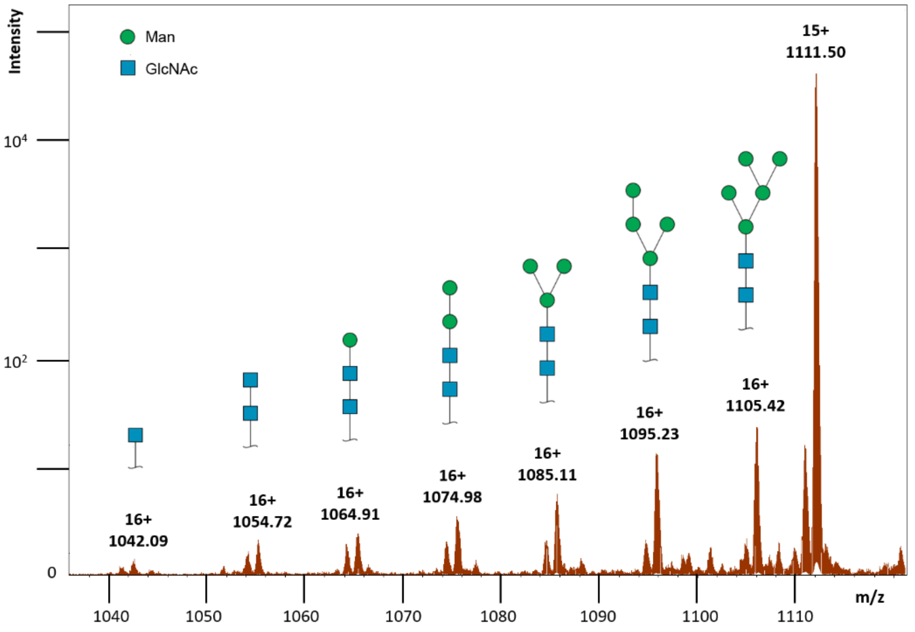

Figure S2. Fragmentation spectrum of *N*-glycans produced by the HEK293S GnTI^{-} cell line. The highest 16× charged peak corresponds to NKp30_Stalk protein with full Asn-GlcNAc$_2$Man$_5$ *N*-glycosylation. Loss of mass in other peaks due to fragmentation corresponds to loss of one mannose unit (rel. mol. mass 162, which is 10.2 on *m*/*z* x-axis with charge z = 16). Loss of mass of the first peak in spectrum from the left corresponds to loss of one *N*-acetylglucosamine unit (rel. mol. mass 203, which is 12.7 on *m*/*z* x-axis with charge z = 16). Predicted oligosaccharide structures corresponding to distinct fragments are depicted by symbolic nomenclature of monosaccharide representation.

As an example of wild-type complex *N*-glycans expressed by the HEK293T cell line, fragmentation spectrum of B7-H6 glycopeptide NMDGTFNVTSSLK is shown below (Figure S3).

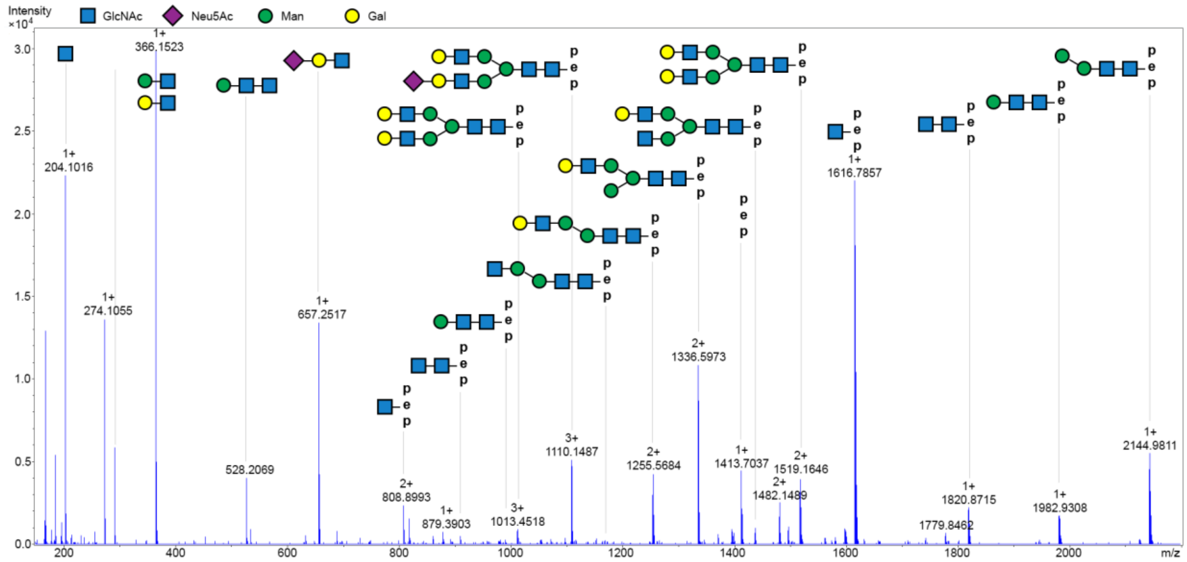

Figure S3. Fragmentation spectrum of *N*-glycans produced by the HEK293T cell line. B7-H6 was digested with trypsin, resultant peptides were desalted and subjected to LC-MS separation and analysis. The fragmentation spectrum of the triply charged precursor with *m*/*z* value of 1110.149 corresponding to peptide NMDGTFNVTSSLK with full Asn-HexNAc$_4$Hex$_5$NeuAc$_1$ *N*-glycosylation is shown together with the predicted oligosaccharide structures corresponding to distinct fragments depicted by symbolic nomenclature of monosaccharide representation.

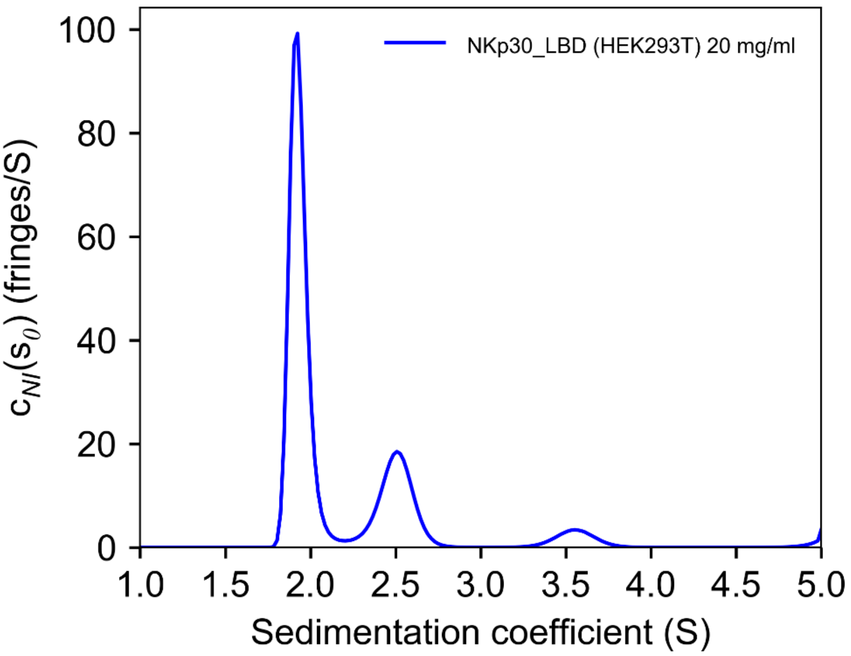

Figure S4. Sedimentation analysis of NKp30_LBD at high concentration. Sample of NKp30_LBD expressed in HEK293T cell line was analyzed by analytical ultracentrifugation at 20 mg/mL concentration using a 3-mm centerpiece and an interference optics. Resultant data were fitted using the nonideal c(s) model implemented in the latest version of Sedfit software [48]. NKp30_LBD is exclusively monomeric at low concentration; however, monomeric, dimeric, and most probably trimeric species are observed at high concentration.

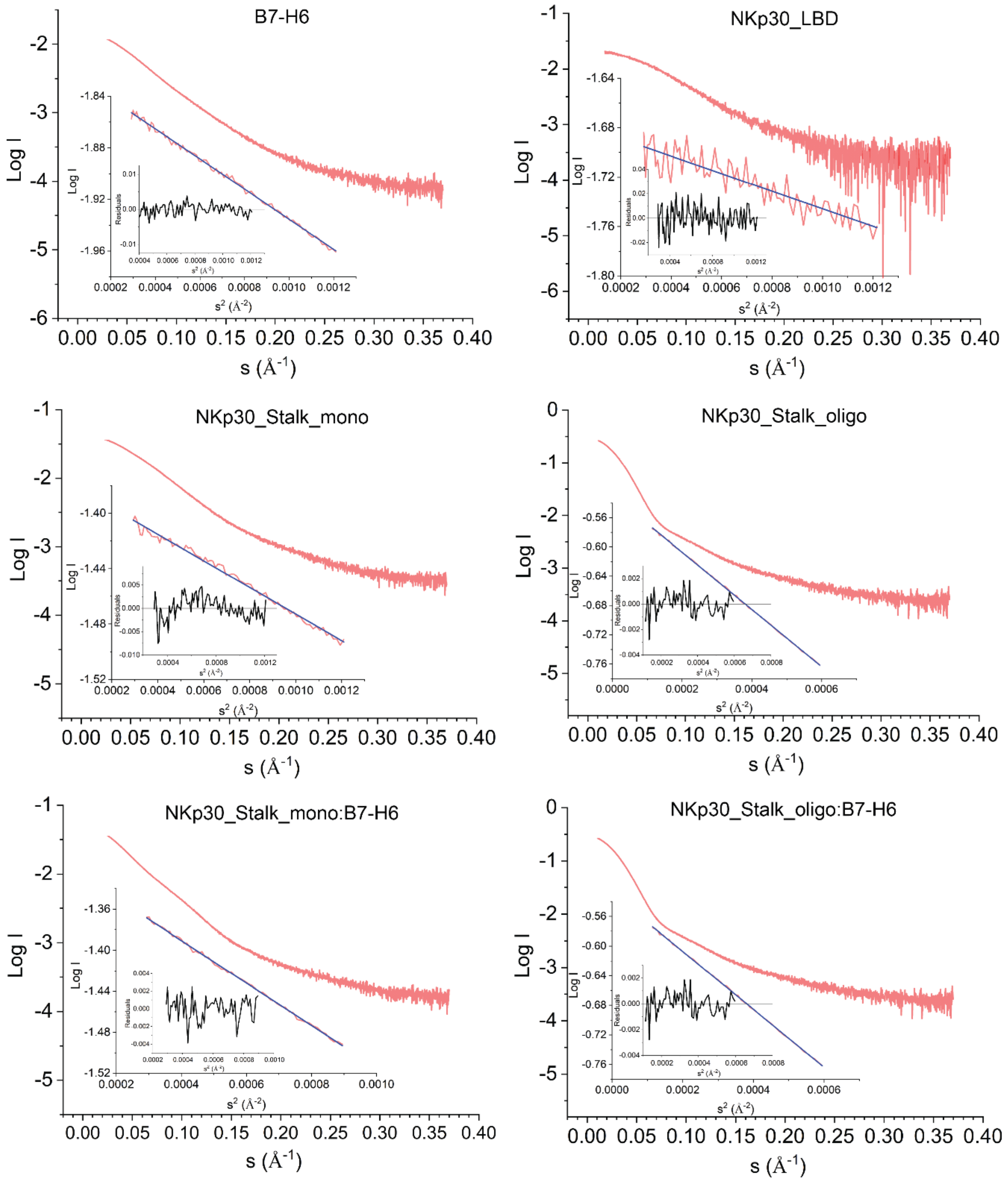

Figure S5. Size exclusion chromatography–small-angle X-ray scattering (SEC-SAXS) data collected for NKp30_LBD, NKp30_Stalk monomeric and oligomeric fractions, B7-H6 and its complex with NKp30_Stalk monomeric and oligomeric fractions. SAXS scattering curves in logarithmic scale (red) are shown for data quality assessment of the merged data, together with the corresponding Guinier plots shown in the lower left corner.

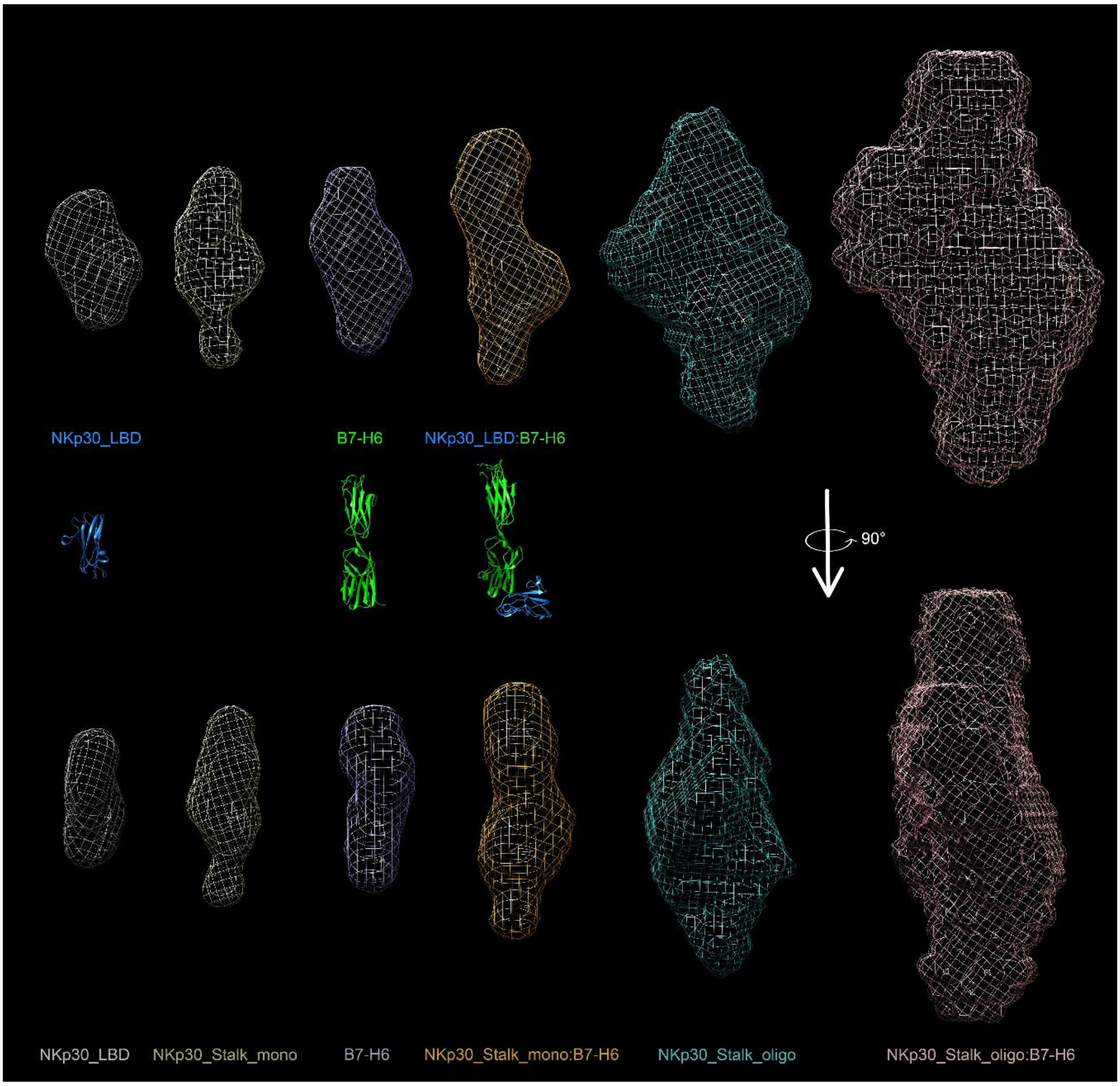

Figure S6. Averaged and filtered DAMMIF envelopes ab initio calculated from the SAXS data for NKp30_LBD, NKp30_Stalk monomeric and oligomeric fractions, B7-H6 and its complex with NKp30_Stalk monomeric and oligomeric fractions. For comparison, NKp30_LBD, B7-H6, and NKp30_LBD:B7-H6 complex, as observed in the presented crystal structure (PDB 6YJP), are shown with the same scale of magnification. The analyzed proteins are glycosylated, and the glycan moieties account for a significant proportion of their molecular mass and hydrodynamic volume; therefore, the envelopes are clearly larger than the naked molecules displayed in the crystal structures.

References

1. Kiessling, R.; Klein, E.; Wigzell, H. „Natural" killer cells in the mouse. I. Cytotoxic cells with specificity for mouse Moloney leukemia cells. Specificity and distribution according to genotype. *Eur. J. Immunol.* 1975, *5*, 112–117, doi:10.1002/eji.1830050208.
2. Herberman, R.B.; Nunn, M.E.; Holden, H.T.; Lavrin, D.H. Natural cytotoxic reactivity of mouse lymphoid cells against syngeneic and allogeneic tumors. II. Characterization of effector cells. *Int. J. Cancer* 1975, *16*, 230–239, doi:10.1002/ijc.2910160205.
3. Pegram, H.J.; Andrews, D.M.; Smyth, M.J.; Darcy, P.K.; Kershaw, M. Activating and inhibitory receptors of natural killer cells. *Immunol. Cell Biol.* 2011, *89*, 216–224, doi:10.1038/icb.2010.78.
4. Kruse, P.H.; Matta, J.; Ugolini, S.; Vivier, E. Natural cytotoxicity receptors and their ligands. *Immunol. Cell Biol.* 2014, *92*, 221–229, doi:10.1038/icb.2013.98.

5. Biassoni, R. Human Natural Killer Receptors, Co-Receptors, and Their Ligands. *Curr. Protoc. Immunol.* 2009, *84*, doi:10.1002/0471142735.im1410s84.
6. Pende, D.; Parolini, S.; Pessino, A.; Sivori, S.; Augugliaro, R.; Morelli, L.; Marcenaro, E.; Accame, L.; Malaspina, A.; Biassoni, R.; et al. Identification and Molecular Characterization of Nkp30, a Novel Triggering Receptor Involved in Natural Cytotoxicity Mediated by Human Natural Killer Cells. *J. Exp. Med.* 1999, *190*, 1505–1516, doi:10.1084/jem.190.10.1505.
7. Memmer, S.; Weil, S.; Beyer, S.; Zöller, T.; Peters, E.; Hartmann, J.; Steinle, A.; Koch, J. The Stalk Domain of NKp30 Contributes to Ligand Binding and Signaling of a Preassembled NKp30-CD3ζ Complex. *J. Biol. Chem.* 2016, *291*, 25427–25438, doi:10.1074/jbc.M116.742981.
8. Kaifu, T.; Escalière, B.; Gastinel, L.N.; Vivier, E.; Baratin, M. B7-H6/NKp30 interaction: A mechanism of alerting NK cells against tumors. *Cell. Mol. Life Sci.* 2011, *68*, 3531–3539, doi:10.1007/s00018-011-0802-7.
9. Hershkovitz, O.; Jarahian, M.; Zilka, A.; Bar-Ilan, A.; Landau, G.; Jivov, S.; Tekoah, Y.; Glicklis, R.; Gallagher, J.T.; Hoffmann, S.C.; et al. Altered glycosylation of recombinant NKp30 hampers binding to heparan sulfate: A lesson for the use of recombinant immunoreceptors as an immunological tool. *Glycobiology* 2008, *18*, 28–41, doi:10.1093/glycob/cwm125.
10. Chisholm, S.E.; Reyburn, H.T. Recognition of Vaccinia Virus-Infected Cells by Human Natural Killer Cells Depends on Natural Cytotoxicity Receptors. *J. Virol.* 2006, *80*, 2225–2233, doi:10.1128/jvi.80.5.2225-2233.2006.
11. Arnon, T.I.; Achdout, H.; Levi, O.; Markel, G.; Saleh, N.; Katz, G.; Gazit, R.; Gonen-Gross, T.; Hanna, J.; Nahari, E.; et al. Inhibition of the NKp30 activating receptor by pp65 of human cytomegalovirus. *Nat. Immunol.* 2005, *6*, 515–523, doi:10.1038/ni1190.
12. Brandt, C.S.; Baratin, M.; Yi, E.C.; Kennedy, J.; Gao, Z.; Fox, B.; Haldeman, B.; Ostrander, C.D.; Kaifu, T.; Chabannon, C.; et al. The B7 family member B7-H6 is a tumor cell ligand for the activating natural killer cell receptor NKp30 in humans. *J. Exp. Med.* 2009, *206*, 1495–1503, doi:10.1084/jem.20090681.
13. Binici, J.; Koch, J. BAG-6, a jack of all trades in health and disease. *Cell. Mol. Life Sci.* 2014, *71*, 1829–1837, doi:10.1007/s00018-013-1522-y.
14. Schlecker, E.; Fiegler, N.; Arnold, A.; Altevogt, P.; Rose-John, S.; Moldenhauer, G.; Sucker, A.; Paschen, A.; Von Strandmann, E.P.; Textor, S.; et al. Metalloprotease-Mediated Tumor Cell Shedding of B7-H6, the Ligand of the Natural Killer Cell-Activating Receptor NKp30. *Cancer Res.* 2014, *74*, 3429–3440, doi:10.1158/0008-5472.can-13-3017.
15. Wang, W.; Guo, H.; Geng, J.; Zheng, X.; Wei, H.; Sun, R.; Tian, Z. Tumor-released Galectin-3, a Soluble Inhibitory Ligand of Human NKp30, Plays an Important Role in Tumor Escape from NK Cell Attack. *J. Biol. Chem.* 2014, *289*, 33311–33319, doi:10.1074/jbc.M114.603464.
16. Chen, Y.; Mo, J.; Jia, X.; He, Y. The B7 Family Member B7-H6: A New Bane of Tumor. *Pathol. Oncol. Res.* 2018, *24*, 717–721, doi:10.1007/s12253-017-0357-5.
17. Hu, Y.; Zeng, T.; Xiao, Z.; Hu, Q.; Li, Y.; Tan, X.; Yue, H.; Wang, W.; Tan, H.; Zou, J. Immunological role and underlying mechanisms of B7-H6 in tumorigenesis. *Clin. Chim. Acta* 2020, *502*, 191–198, doi:10.1016/j.cca.2019.12.030.
18. Matta, J.; Baratin, M.; Chiche, L.; Forel, J.-M.; Cognet, C.; Thomas, G.; Farnarier, C.; Piperoglou, C.; Papazian, L.; Chaussabel, D.; et al. Induction of B7-H6, a ligand for the natural killer cell–activating receptor NKp30, in inflammatory conditions. *Blood* 2013, *122*, 394–404, doi:10.1182/blood-2013-01-481705.
19. Joyce, M.G.; Tran, P.; Zhuravleva, M.A.; Jaw, J.; Colonna, M.; Sun, P.D. Crystal structure of human natural cytotoxicity receptor NKp30 and identification of its ligand binding site. *Proc. Natl. Acad. Sci. USA* 2011, *108*, 6223–6228, doi: 10.1073/pnas.1100622108.
20. Li, Y.; Wang, Q.; Mariuzza, R.A. Structure of the human activating natural cytotoxicity receptor NKp30 bound to its tumor cell ligand B7-H6. *J. Exp. Med.* 2011, *208*, 703–714, doi:10.1084/jem.20102548.
21. Herrmann, J.; Berberich, H.; Hartmann, J.; Beyer, S.; Davies, K.E.; Koch, J. Homo-oligomerization of the Activating Natural Killer Cell Receptor NKp30 Ectodomain Increases Its Binding Affinity for Cellular Ligands. *J. Biol. Chem.* 2014, *289*, 765–777, doi:10.1074/jbc.m113.514786.
22. Hartmann, J.; Tran, T.-V.; Kaudeer, J.; Oberle, K.; Herrmann, J.; Quagliano, I.; Abel, T.; Cohnen, A.; Gatterdam, V.; Jacobs, A.; et al. The Stalk Domain and the Glycosylation Status of the Activating Natural Killer Cell Receptor NKp30 Are Important for Ligand Binding. *J. Biol. Chem.* 2012, *287*, 31527–31539, doi:10.1074/jbc.m111.304238.

23. Binici, J.; Hartmann, J.; Herrmann, J.; Schreiber, C.; Beyer, S.; Güler, G.; Vogel, V.; Tumulka, F.; Abele, R.; Mäntele, W.; et al. A Soluble Fragment of the Tumor Antigen BCL2-associated Athanogene 6 (BAG-6) Is Essential and Sufficient for Inhibition of NKp30 Receptor-dependent Cytotoxicity of Natural Killer Cells. *J. Biol. Chem.* 2013, *288*, 34295–34303, doi:10.1074/jbc.M113.483602.
24. Aricescu, A.R.; Lu, W.; Jones, E.Y. A time- and cost-efficient system for high-level protein production in mammalian cells. *Acta Crystallogr. Sect. D Biol. Crystallogr.* 2006, *62*, 1243–1250, doi:10.1107/s0907444906029799.
25. Reeves, P.J.; Callewaert, N.; Contreras, R.; Khorana, H.G. Structure and function in rhodopsin: High-level expression of rhodopsin with restricted and homogeneous N-glycosylation by a tetracycline-inducible N-acetylglucosaminyltransferase I-negative HEK293S stable mammalian cell line. *Proc. Natl. Acad. Sci. USA* 2002, *99*, 13419–13424.
26. Bláha, J.; Maraun, M.; Novák, P.; Vaněk, O. Expression and purification of soluble and stable ectodomain of natural killer cell receptor LLT1 through high-density transfection of suspension adapted HEK293S GnTI− cells. *Protein Expr. Purif.* 2015, *109*, 7–13, doi:10.1016/j.pep.2015.01.006.
27. Krissinel, E.; Henrick, K. Inference of Macromolecular Assemblies from Crystalline State. *J. Mol. Biol.* 2007, *372*, 774–797, doi:10.1016/j.jmb.2007.05.022.
28. Ni, L.; Dong, C. New B7 Family Checkpoints in Human Cancers. *Mol. Cancer Ther.* 2017, *16*, 1203–1211, doi:10.1158/1535-7163.MCT-16-0761.
29. Wu, M.-R.; Zhang, T.; Gacerez, A.T.; Coupet, T.A.; Demars, L.R.; Sentman, C.L. B7H6-Specific Bispecific T Cell Engagers Lead to Tumor Elimination and Host Antitumor Immunity. *J. Immunol.* 2015, *194*, 5305–5311, doi:10.4049/jimmunol.1402517.
30. Gacerez, A.T.; Hua, C.K.; Ackerman, M.E.; Sentman, C.L. Chimeric antigen receptors with human scFvs preferentially induce T cell anti-tumor activity against tumors with high B7H6 expression. *Cancer Immunol. Immunother.* 2018, *67*, 749–759, doi:10.1007/s00262-018-2124-1.
31. Kellner, C.; Maurer, T.; Hallack, D.; Repp, R.; Van De Winkel, J.G.J.; Parren, P.W.H.I.; Valerius, T.; Humpe, A.; Gramatzki, M.; Peipp, M.; et al. Mimicking an Induced Self Phenotype by Coating Lymphomas with the NKp30 Ligand B7-H6 Promotes NK Cell Cytotoxicity. *J. Immunol.* 2012, *189*, 5037–5046, doi:10.4049/jimmunol.1201321.
32. Kellner, C.; Günther, A.; Humpe, A.; Repp, R.; Klausz, K.; Derer, S.; Valerius, T.; Ritgen, M.; Brüggemann, M.; Van De Winkel, J.G.; et al. Enhancing natural killer cell-mediated lysis of lymphoma cells by combining therapeutic antibodies with CD20-specific immunoligands engaging NKG2D or NKp30. *OncoImmunology* 2016, *5*, e1058459, doi:10.1080/2162402X.2015.1058459.
33. Peipp, M.; Derer, S.; Lohse, S.; Staudinger, M.; Klausz, K.; Valerius, T.; Gramatzki, M.; Kellner, C. HER2-specific immunoligands engaging NKp30 or NKp80 trigger NK-cell-mediated lysis of tumor cells and enhance antibody-dependent cell-mediated cytotoxicity. *Oncotarget* 2015, *6*, 32075–32088, doi:10.18632/oncotarget.5135.
34. Jaron-Mendelson, M.; Yossef, R.; Appel, M.; Zilka, A.; Hadad, U.; Afergan, F.; Rosental, B.; Engel, S.; Nedvetzki, S.; Braiman, A.; et al. Dimerization of NKp46 Receptor Is Essential for NKp46-Mediated Lysis: Characterization of the Dimerization Site by Epitope Mapping. *J. Immunol.* 2012, *188*, 6165–6174, doi:10.4049/jimmunol.1102496.
35. Hadad, U.; Thauland, T.J.; Martinez, O.M.; Butte, M.J.; Porgador, A.; Krams, S.M. NKp46 Clusters at the Immune Synapse and Regulates NK Cell Polarization. *Front. Immunol.* 2015, *6*, 216, doi:10.3389/fimmu.2015.00495.
36. Arnon, T.I.; Markel, G.; Bar-Ilan, A.; Hanna, J.H.; Fima, E.; Benchetrit, F.; Galili, R.; Cerwenka, A.; Benharroch, D.; Sion-Vardy, N.; et al. Harnessing Soluble NK Cell Killer Receptors for the Generation of Novel Cancer Immune Therapy. *PLoS ONE* 2008, *3*, e2150, doi:10.1371/journal.pone.0002150.
37. Franke, D.; Petoukhov, M.V.; Konarev, P.V.; Panjkovich, A.; Tuukkanen, A.; Mertens, H.D.T.; Kikhney, A.G.; Hajizadeh, N.R.; Franklin, J.M.; Jeffries, C.M.; et al. ATSAS 2.8: A comprehensive data analysis suite for small-angle scattering from macromolecular solutions. *J. Appl. Crystallogr.* 2017, *50*, 1212–1225, doi:10.1107/s1600576717007786.
38. Pettersen, E.F.; Goddard, T.D.; Huang, C.C.; Couch, G.S.; Greenblatt, D.M.; Meng, E.C.; Ferrin, T.E. UCSF Chimera–a visualization system for exploratory research and analysis. *J. Comput. Chem.* 2004, *25*, 1605–1612.

39. Xu, X.; Li, Y.; Gauthier, L.; Chen, Q.; Vivier, E.; Mariuzza, R.A. Expression, crystallization and X-ray diffraction analysis of a complex between B7-H6, a tumor cell ligand for the natural cytotoxicity receptor NKp30, and an inhibitory antibody. *Acta Crystallogr. Sect. F Struct. Biol. Commun.* 2015, *71*, 697–701, doi:10.1107/s2053230x15006755.
40. Xu, X.; Narni-Mancinelli, E.; Cantoni, C.; Li, Y.; Guia, S.; Gauthier, L.; Chen, Q.; Moretta, A.; Vély, F.; Eisenstein, E.; et al. Structural Insights into the Inhibitory Mechanism of an Antibody against B7-H6, a Stress-Induced Cellular Ligand for the Natural Killer Cell Receptor NKp30. *J. Mol. Biol.* 2016, *428*, 4457–4466, doi:10.1016/j.jmb.2016.09.011.
41. Durocher, Y. High-level and high-throughput recombinant protein production by transient transfection of suspension-growing human 293-EBNA1 cells. *Nucleic Acids Res.* 2002, *30*, 9e, doi:10.1093/nar/30.2.e9.
42. Backliwal, G.; Hildinger, M.; Kuettel, I.; Delegrange, F.; Hacker, D.L.; Wurm, F.M. Valproic acid: A viable alternative to sodium butyrate for enhancing protein expression in mammalian cell cultures. *Biotechnol. Bioeng.* 2008, *101*, 182–189, doi:10.1002/bit.21882.
43. Pompach, P.; Man, P.; Kavan, D.; Hofbauerová, K.; Kumar, V.; Bezouska, K.; Havlicek, V.; Novák, P. Modified electrophoretic and digestion conditions allow a simplified mass spectrometric evaluation of disulfide bonds. *J. Mass Spectrom.* 2009, *44*, 1571–1578, doi:10.1002/jms.1609.
44. Young, M.M.; Tang, N.; Hempel, J.C.; Oshiro, C.M.; Taylor, E.W.; Kuntz, I.D.; Gibson, B.W.; Dollinger, G. High throughput protein fold identification by using experimental constraints derived from intramolecular cross-links and mass spectrometry. *Proc. Natl. Acad. Sci. USA* 2000, *97*, 5802–5806.
45. Kukačka, Z.; Rosulek, M.; Strohalm, M.; Kavan, D.; Novák, P. Mapping protein structural changes by quantitative cross-linking. *Methods* 2015, *89*, 112–120, doi:10.1016/j.ymeth.2015.05.027.
46. Grüninger, F.; D'Arcy, A.; D'Arcy, B.; Chène, C. Deglycosylation of proteins for crystallization using recombinant fusion protein glycosidases. *Protein Sci.* 1996, *5*, 2617–2622, doi:10.1002/pro.5560051224.
47. Schuck, P. Size-Distribution Analysis of Macromolecules by Sedimentation Velocity Ultracentrifugation and Lamm Equation Modeling. *Biophys. J.* 2000, *78*, 1606–1619, doi:10.1016/s0006-3495(00)76713-0.
48. Chaturvedi, S.; Ma, J.; Brown, P.H.; Zhao, H.; Schuck, P. Measuring macromolecular size distributions and interactions at high concentrations by sedimentation velocity. *Nat. Commun.* 2018, *9*, 4415, doi:10.1038/s41467-018-06902-x.
49. Scheuermann, T.H.; Brautigam, C.A. High-precision, automated integration of multiple isothermal titration calorimetric thermograms: New features of NITPIC. *Methods* 2015, *76*, 87–98, doi:10.1016/j.ymeth.2014.11.024.
50. Zhao, H.; Piszczek, G.; Schuck, P. SEDPHAT--a platform for global ITC analysis and global multi-method analysis of molecular interactions. *Methods* 2015, *76*, 137–148, doi:10.1016/j.ymeth.2014.11.012.
51. Brautigam, C.A. Calculations and Publication-Quality Illustrations for Analytical Ultracentrifugation Data. *Methods Enzymol.* 2015, *562*, 109–133, doi:10.1016/bs.mie.2015.05.001.
52. Kabsch, W. XDS. *Acta Crystallogr. Sect. D Biol. Crystallogr.* 2010, *66*, 125–132, doi:10.1107/S0907444909047337.
53. Winn, M.; Ballard, C.C.; Cowtan, K.; Dodson, E.J.; Emsley, P.; Evans, P.R.; Keegan, R.M.; Krissinel, E.B.; Leslie, A.G.W.; McCoy, A.; et al. Overview of the CCP4 suite and current developments. *Acta Crystallogr. Sect. D Biol. Crystallogr.* 2011, *67*, 235–242, doi:10.1107/S0907444910045749.
54. Vagin, A.; Lebedev, A. MoRDa, an automatic molecular replacement pipeline. *Acta Crystallogr. Sect. A Found. Adv.* 2015, *71*, S19, doi:10.1107/s2053273315099672.
55. McCoy, A.J.; Grosse-Kunstleve, R.W.; Adams, P.D.; Winn, M.D.; Storoni, L.C.; Read, R.J. Phaser crystallographic software. *J. Appl. Crystallogr.* 2007, *40*, 658–674, doi:10.1107/S0021889807021206.
56. Murshudov, G.N.; Skubak, P.; Lebedev, A.A.; Pannu, N.S.; Steiner, R.A.; Nicholls, R.; Winn, M.D.; Long, F.; Vagin, A.A. REFMAC5 for the refinement of macromolecular crystal structures. *Acta Crystallogr. Sect. D Biol. Crystallogr.* 2011, *67*, 355–367, doi:10.1107/S0907444911001314.
57. Kovalevskiy, O.; Nicholls, R.; Murshudov, G. Automated refinement of macromolecular structures at low resolution using prior information. *Acta Crystallogr. Sect. D Struct. Biol.* 2016, *72*, 1149–1161, doi:10.1107/S2059798316014534.
58. Emsley, P.; Lohkamp, B.; Scott, W.G.; Cowtan, K. Features and development of Coot. *Acta Crystallogr. Sect. D Biol. Crystallogr.* 2010, *66*, 486–501, doi:10.1107/S0907444910007493.

59. Chen, V.B.; Arendall, W.B.; Headd, J.J.; Keedy, D.; Immormino, R.M.; Kapral, G.J.; Murray, L.W.; Richardson, J.S.; Richardson, D.C. MolProbity: All-atom structure validation for macromolecular crystallography. *Acta Crystallogr. Sect. D Biol. Crystallogr.* 2010, *66*, 12–21, doi:10.1107/S0907444909042073.

© 2020 by the authors. Licensee MDPI, Basel, Switzerland. This article is an open access article distributed under the terms and conditions of the Creative Commons Attribution (CC BY) license (http://creativecommons.org/licenses/by/4.0/).
